# Diabetes Differentially Affects Vascular Reactivity in Isolated Human Arterial and Venous Bypass Grafts

**DOI:** 10.3390/life15030454

**Published:** 2025-03-13

**Authors:** Aylin Vidin Şen, Birsel Sönmez Uydeş Doğan, Uğur Kısa, Cevdet Uğur Koçoğulları, Önder Teskin, Fatoş İlkay Alp Yıldırım

**Affiliations:** 1 Department of Pharmacology, Faculty of Pharmacy, İstanbul University, 34116 İstanbul, Turkey; aylin.vidinsen@bau.edu.tr (A.V.Ş.); sonmezdo@istanbul.edu.tr (B.S.U.D.); 2 Department of Pharmacology, School of Pharmacy, Bahçeşehir University, 34351 İstanbul, Turkey; 3 Department of Cardiovascular Surgery, Dr. Siyami Ersek Thoracic and Cardiovascular Surgery Training and Research Hospital, 34668 İstanbul, Turkey; drugurkisa@gmail.com (U.K.); cevdetkocogullari@gmail.com (C.U.K.); 4 Department of Cardiovascular Surgery, Faculty of Medicine, Biruni University, 34010 İstanbul, Turkey; oteskin@gmail.com

**Keywords:** diabetes mellitus, internal mammary artery, saphenous vein, coronary artery bypass surgery, vascular reactivity

## Abstract

Arterial and venous graft spasm can occur during harvesting or immediately after coronary artery bypass grafting (CABG), leading to increased perioperative morbidity and affecting graft patency rates. Bypass grafts harvested from diabetic patients are particularly prone to spasm. This study aimed to elucidate the functional characteristics of human bypass grafts for the internal mammary artery (IMA) and saphenous vein (SV), from both diabetic and non-diabetic patients, and to determine how diabetes affected their responses to spasmogenic and relaxant agents. SV and IMA graft rings isolated from diabetic and non-diabetic patients during CABG were placed in an isolated organ bath system. Contractions to potassium chloride (10–100 mM) and phenylephrine (10^−8^–10^−4^ M) were evaluated, and relaxation responses to acetylcholine (10^−9^–10^−4^ M) and sodium nitroprusside (10^−8^–10^−4^ M) were assessed to evaluate endothelial and smooth muscle function, respectively. We observed increased responses to phenylephrine, an alpha-1 adrenoceptor agonist, in both IMAs and SVs, as well as an increased responses to potassium chloride, a non-receptor agonist, in SVs in diabetic patients compared to non-diabetic patients. We did not observe any deterioration in endothelium-dependent relaxations in either SV or IMA grafts under diabetic conditions. This study is the first to demonstrate that diabetes exacerbates potassium chloride-induced contractions in human SV grafts. Understanding the differences in potassium chloride-induced contraction profiles between arterial and venous grafts is essential in optimizing graft spasm management and improving the patency rates of bypass grafts.

## 1. Introduction

Coronary artery bypass grafting (CABG) is a surgical procedure used to treat patients with coronary artery disease [1]. The saphenous vein (SV) is commonly used as a conduit for CABG surgery because of its ready accessibility and being easy to handle [2]. Despite its advantages, the SV has a poor patency rate due to early thrombosis and intimal hyperplasia [3], whereas the internal mammary artery (IMA) is the most frequently used arterial graft material and has 90% long-term patency rates over 10 years and high survival benefits [4].

The incidence of diabetes mellitus (DM) in patients undergoing CABG surgery continues to rise, and it is now estimated that more than 30–40% of CABG patients have DM or metabolic syndrome [5]. Although numerous studies have shown comparable graft patency rates in both diabetic and non-diabetic patients [6,7,8], additional studies have shown better patency rates in non-diabetic patients, particularly those revascularized with SV grafts [9,10]. This discrepancy indicates that DM may differentially impact graft outcomes, though the specific mechanisms remain unclear.

A significant complication that affects graft patency in CABG is arterial and venous graft spasm, which can occur during graft harvesting or immediately post-surgery. This spasm can lead to increased perioperative morbidity and premature occlusion [11], as well as reduced graft patency rates [12]. Bypass grafts harvested from patients with DM are more prone to spasm after implantation into the coronary circulation [13]. DM increases the activity or sensitivity of the vascular smooth muscle to various vasoconstrictors [14]. The increased reactivity of vascular smooth muscle in DM is thought to be due to either impaired endothelium-dependent vasodilation or enhanced vascular contractility due to endothelium-independent mechanisms [15]. These factors may directly contribute to acute vascular dysfunction and impact long-term survival outcomes [16].

Despite the established relationship between DM and graft complications, the effects of diabetes on the functional characteristics of different bypass grafts, such as IMA and SV grafts, remain poorly understood. The exact purpose of this study was to explore this unclarified point by examining the contractile and relaxant responses of arterial and venous bypass grafts, specifically IMA and SV grafts, in both diabetic (DM+) and non-diabetic (DM−) patients, and to draw attention to factors influence vascular reactivity in the perioperative and/or postoperative periods. Thus, we determined how DM influences the contractile responses to receptor-dependent (alpha-1 receptor agonist, Phe) and -independent spasmogenic agents (depolarizing agent, KCl) and the relaxant responses that are endothelium-dependent and -independent. Understanding these effects will provide crucial insights into the management of CABG patients with diabetes and potentially inform surgical and pharmacological strategies to improve graft patency and patient outcomes.

## 2. Materials and Methods

### 2.1. Ethical Declaration

The study was approved by the Institutional Review Board of Haydarpasa Numune Training and Research Hospital, Istanbul, Turkey (Protocol Number: HNEAH-KAEK 2020/KK/224; Date of Approval, 23 December 2020). All the patients voluntarily participated and provided informed written consent. The study followed the principles of the Declaration of Helsinki.

### 2.2. Patient Population

SV and IMA grafts were obtained from patients undergoing CABG surgery at Dr. Siyami Ersek Thoracic and Cardiovascular Surgery Education Research Hospital and Biruni University Hospital, Istanbul, Turkey. The inclusion criteria for our study were as follows: • Patients had undergone coronary bypass surgery. • All participants were over 18 years of age. Patients were divided into two groups: those with a diagnosis of diabetes mellitus (DM+) and those without diabetes (DM−), classified as normoglycemic.

The demographic and pre-operative characteristics, as well as the numbers of patients, are shown in Table 1. From each bypass graft sample, 2 to 4 specimens were prepared, and the “n” numbers used for each experimental setting are indicated in the related figure legends.

Caution was taken during harvesting to avoid stretching the vessel and touching the endothelial surface. The sampled segments of the IMA and SV, which were not exposed to any preparation solution, were placed into cold (4 °C) Krebs-Ringer–bicarbonate solution (in mM): NaCl 118.5, KCl 4.8, KH_2_PO_4_ 1.2, NaHCO_3_ 25, MgSO_4_•7H_2_O 1.2, CaCl_2_ 1.9, glucose 5.5, and disodium EDTA 0.026, then immediately transferred to the laboratory. The vessels were carefully cleaned of excess fat and connective tissues and then cut into 2–4 mm rings. In order to evaluate the vascular reactivity, the isometric force (Grass FT03 transducer, Quincy, MA, USA) was measured in 10 mL organ baths (Powerlab ADInstruments^®^, Colorado Springs, CO, USA) filled with the Krebs-Ringer–bicarbonate solution at 37 °C and gassed with 95%O_2_–5%CO_2_ (pH = 7.4). The resting tension was adjusted to 1 g for the IMA (~1 h) and 2 g for the SV (~2 h) during the equilibration period, and the organ bath solution was refreshed every 15 min.

Rings were challenged by two consecutive potassium chloride (KCl, 40 mM) to check the viability and provide the standardization of the vessels. Thereafter, vasoconstrictor responses of the vessels were assessed by generating concentration–response curves to the α-adrenergic receptor agonist, phenylephrine (Phe; 10^−8^–10^−4^ M), and non-receptor agonist, KCl (10 mM–100 mM). The endothelium- and smooth muscle-dependent relaxation capacity of the vessels were checked by acetylcholine (ACh; 10^−9^–10^−4^ M) and the nitrovasodilator, sodium nitroprusside (SNP; 10^−9^–10^−4^ M), respectively, following precontraction with Phe (3 × 10^−6^–10^−5^ M).

### 2.3. Chemicals

Phenylephrine was purchased from Cayman Chemical (Ann Arbor, MI, USA), and potassium chloride was purchased from Merck (Darmstadt, Germany), while sodium nitroprusside (and acetylcholine) were obtained from Sigma-Aldrich (St. Louis, MO, USA).

### 2.4. Statistical Analysis

Data are presented as “mean ± S.E.M”, and “n” refers to the number of patients from whom vessels were obtained. The contractile responses to KCl and Phe are expressed as “g” and “% of KCl 40 mM” contraction, whereas the relaxant responses to ACh and SNP are indicated as % decreases in the precontractile tone. This approach provides both absolute force measurements and relative responses, ensuring a comprehensive evaluation of vascular reactivity. Maximal responses (contraction and relaxation) are expressed as Emax and sensitivities of the vessels (pEC50) to Phe, and ACh and SNP were calculated as the effective concentration that elicits 50% of the maximal response by using non-linear regression curve fitting and are expressed as −log EC50. Statistical analyses were determined by Student’s *t*-test, as well as by two-way analysis of variance (ANOVA) followed by Bonferroni’s post hoc test where appropriate. A “*p*” value <0.05 was considered as statistically significant. Statistical analyses were performed by using GraphPad PRISM 5.0 (GraphPad Software Inc., San Diego, CA, USA).

## 3. Results

### 3.1. Contractile Responses of Internal Mammary Arteries (IMAs) and Saphenous Veins (SVs) Isolated from Diabetic (DM+) and Non-Diabetic (DM−) Patients

**Figure 1:**
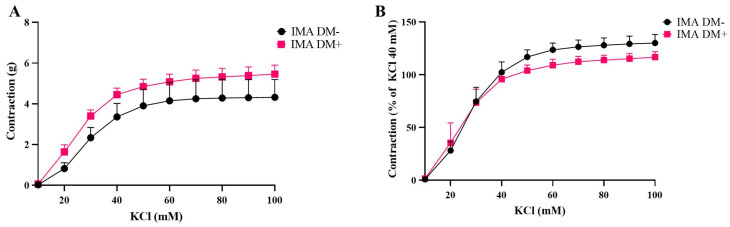
Cumulative concentration–response curves for potassium chloride (KCl, 10–100 mM) in isolated human internal mammary arteries (IMAs) obtained from diabetic (DM+, n = 7) and non-diabetic patients (DM−, n = 5). Responses are expressed as “g” (**A**) and % of KCI 40 mM (**B**). Values are given as mean ± S.E.M.

The cumulative administration of potassium chloride (10–100 mM) induced contractile responses in human IMA grafts in a concentration-dependent manner both in the DM+ (n = 7) and DM− groups (n = 5) (Figure 1). There were no significant differences between the DM+ and DM− groups in terms of potassium chloride-induced contractions in the isolated IMA grafts (Figure 1).

**Figure 2:**
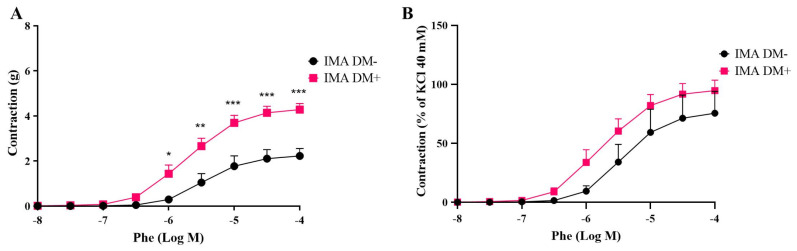
Cumulative concentration–response curves of phenylephrine (Phe, 10^−8^–10^−4^ M) in isolated human internal mammary arteries (IMAs) obtained from diabetic (DM+, n = 7) and non-diabetic patients (DM−, n = 5). Responses are expressed as “g” (**A**) and % of KCI 40 mM (**B**). Values are given as mean ± S.E.M. * *p* < 0.05; ** *p* < 0.01; *** *p* < 0.001.

On the other hand, phenylephrine-induced contractions were significantly increased particularly at high concentrations in DM+ patients (Figure 2).

**Figure 3:**
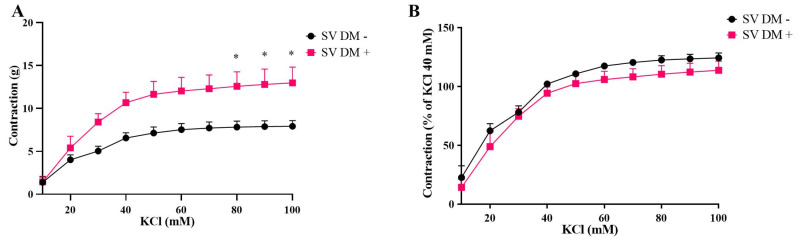
Cumulative concentration–response curves for potassium chloride (10–100 mM) in isolated human saphenous veins (SVs) obtained from diabetic (DM+, n = 5) and non-diabetic patients (DM−, n = 5). Responses are expressed as “g” (**A**) and as % of KCI 40 mM (**B**). Values are given as mean ± S.E.M. * *p* < 0.05.

**Figure 4:**
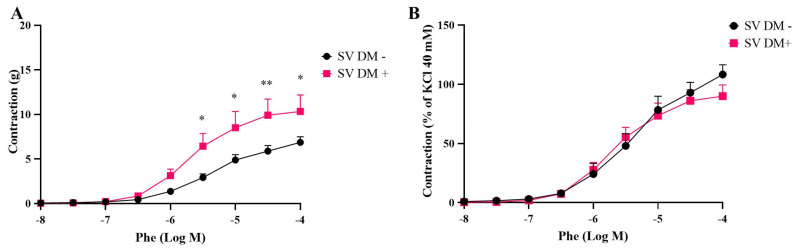
Cumulative concentration–response curves for phenylephrine (Phe, 10^−8^–10^−4^ M) in isolated human saphenous veins (SVs) obtained from diabetic (DM+, n = 5) and non-diabetic patients (DM−, n = 5). Responses are expressed as “g” (**A**) and % of KCI 40 mM (**B**). Values are given as mean ± S.E.M. * *p* < 0.05; ** *p* < 0.01.

Interestingly, in SV grafts, both potassium chloride- and phenylephrine-induced contractions were significantly augmented in DM+ patients compared to DM− patients (Figure 3 and Figure 4).

Maximal contractile responses (Emax) and the sensitivity (pEC50) to spasmogens in isolated IMA and SV samples are presented in Table 2.

### 3.2. Endothelium-Dependent and Endothelium-Independent Relaxant Responses of Internal Mammary Arteries (IMAs) and Saphenous Veins (SVs) Isolated from Diabetic (DM+) and Non-Diabetic (DM−) Patients

**Figure 5:**
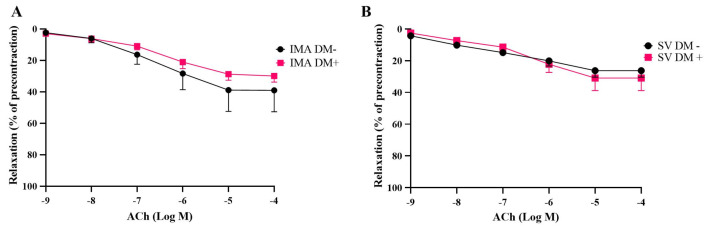
Cumulative concentration–response curves for acetylcholine (ACh; 10^−9^–10^−4^ M) in human internal mammary arteries (IMAs) (**A**) and saphenous veins (SVs) (**B**) isolated from diabetic [DM+, n = 7 (IMAs), n = 5 (SVs)] and non-diabetic patients [DM−, n = 5 (IMAs), n = 5 (SVs)]. Responses are expressed as a % decrease in the precontractile tone induced by a submaximal concentration of Phe (3 × 10^−6^–10^−5^ M).

**Figure 6:**
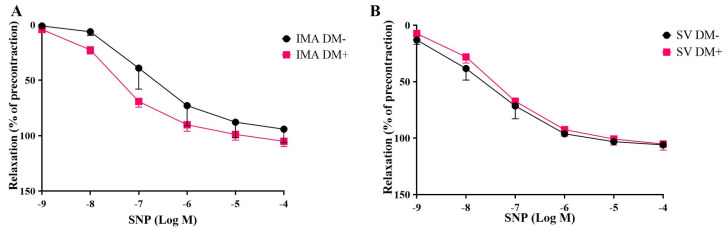
Cumulative concentration–response curves for sodium nitroprusside (SNP; 10^−9^–10^−4^ M) in human internal mammary arteries (IMAs) (**A**) and saphenous veins (SVs) (**B**) isolated from diabetic [DM+, n = 7 (IMAs), n = 5 (SVs)] and non-diabetic patients [DM−, n = 5 (IMAs), n = 5 (SVs)]. Responses are expressed as a % decrease in the precontractile tone induced by a submaximal concentration of Phe (3 × 10^−6^–10^−5^ M).

The endothelium-dependent relaxing agent acetylcholine and the endothelium-independent vasodilator sodium nitroprusside produced concentration-dependent relaxations in both human SV and IMA rings isolated form DM+ and DM− patients (Figure 5 and Figure 6).

The presence of diabetes did not lead to any significant changes in the responses to acetylcholine (Figure 5) and sodium nitroprusside (Figure 6) in either IMA or SV grafts in terms of Emax and pEC50 values (Table 3).

## 4. Discussion

Diabetes negatively impacts short- and long-term survival rates and increases the incidence of ischemic events, particularly after myocardial revascularization [17]. Bypass grafts harvested from diabetic patients are more prone to vasospasm after implantation into the coronary circulation [13]. Postoperative outcomes, especially in the initial stages, can be significantly influenced by the vascular responsiveness of the bypass conduits [17]. The underlying mechanisms behind diabetic vascular smooth muscle hyperreactivity are generally considered to be related to either impaired endothelium-dependent vasodilation or enhanced contractility in the vessels [15]. Conduit properties may directly contribute to acute term vascular dysfunction and long-term survival [16]. Several studies assessing the vasoreactivity of bypass grafts, with SVs and IMAs, isolated from both diabetic (DM+) and non-diabetic (DM−) patients, however, displayed inconsistent results. Herein, our findings explore the impact of diabetes on the vascular responsiveness of isolated human venous (SV) and arterial (IMA) bypass grafts to spasmogenic and relaxant agents.

This study examined the impact of diabetes on the vascular responsiveness of isolated human venous (saphenous vein, SV) and arterial (internal mammary artery, IMA) bypass grafts to spasmogenic and relaxant agents. Our results showed that potassium chloride-induced contractile responses in IMA grafts were not found to be different between the DM+ and DM− groups, which is consistent with previous findings in studies conducted by Lorusso et al. [17] and Wendler et al. [18]. In contrast, several studies demonstrated an augmented response to potassium chloride in IMA grafts isolated from diabetic patients [19,20,21]. Regarding SV, our results demonstrated that potassium chloride-induced contractions were significantly enhanced in SV grafts isolated from DM+ patients compared to DM− patients. In contrary, previous studies indicated no differences in terms of potassium chloride-induced contractions between SV grafts obtained from DM+ and DM− patients [17,22]. To the best of our knowledge, this is the first study demonstrating the influence of diabetic conditions on potassium chloride-induced contractions in SVs.

The morphological and physiological distinctions between arteries and veins are expected to lead to significant differences in the vascular reactivity of coronary artery bypass grafts [23]. Potassium chloride induces contraction due to membrane depolarization via calcium entry through voltage-operated calcium channels. Some studies suggest a decrease or no change, whereas the majority of research indicates a notable rise in L-type calcium channel activity in the vascular smooth muscle in various experimental diabetic models and in the presence of hyperglycemia [24,25,26,27,28]. These differences can be attributed to several factors, including the use of diverse vascular segments and cultured versus freshly isolated cells [24]. Bieger et al. reported that in human SV, L-type calcium channels have low threshold activation [29]. Although no data are available concerning this situation in diabetes, the report by Bieger et al. may explain why potassium chloride-induced contractions changed in SV grafts but not in IMA grafts isolated from DM+ patients in our experimental settings. In diabetic conditions, there is evidence that L-type calcium channels are upregulated [30], leading to a greater calcium influx upon membrane depolarization. This enhanced calcium entry could explain the stronger contractile responses to KCl observed in the SV grafts compared to non-diabetic vessels.

Another possible mechanism that could explain the differences in potassium chloride-induced responses could be calcium sensitization. Potassium chloride can cause calcium sensitization via the activation of the RhoA-Rho kinase pathway [31], which is involved in many cellular functions and the pathogenesis of cardiovascular diseases and DM [32]. Reports regarding the influence of DM on RhoA activity and/or expression are inconsistent. Kun et al. reported that the expression level of RhoA and Rho kinase mRNA in SVs was not significantly different from IMA grafts but showed a rising trend in SVs compared to IMAs [33]. Moreover, Ding et al. indicated that RhoA expression was significantly increased in SVs from diabetic patients, whereas in IMA and radial artery grafts, no difference was observed between diabetic and non-diabetic patients [34]. In addition, Riches et al. found that DM exhibited impairment in smooth muscle cell function in human SVs due to changes in the expression and activity of RhoA, which was not observed in IMAs [32]. Therefore, diabetes-induced variations in calcium-dependent and -independent mechanisms regulating vascular tone in SV grafts may account for the differences observed in our study regarding potassium chloride-induced contractions between SV and IMA grafts. Further studies are needed to determine the underlying mechanisms mediating potassium chloride-induced hyperreactivity in SVs isolated from diabetic patients.

Our results revealed that phenylephrine-induced contractions were significantly higher in both SV and IMA grafts taken from diabetic patients compared to non-diabetics, which is consistent with earlier studies [20,21]. This finding could be explained by a diabetes-induced increase in the expression and/or activity of alpha-1 adrenoceptor in both SVs and IMAs [20,35]. Diabetic vessels displayed augmented vasoconstriction due to either endothelial dysfunction or the increased contractility of smooth muscle cells [35] through receptor-dependent and receptor-independent mechanisms [20]. Although it is well known that diabetes and hyperglycemia exacerbate endothelial dysfunction attributed to a decrease in nitric oxide (NO) bioavailability, as well as a reduced response from vascular smooth muscle cells to NO [36], we did not observe any deterioration in acetylcholine-mediated endothelium-dependent relaxation responses in either SV or IMA grafts under diabetic conditions. Thus, our results suggest that the increased contractility to spasmogens may not be associated with endothelial dysfunction, as endothelium-mediated relaxations did not differ between diabetic and non-diabetic patients, consistent with previous studies [18,34]. However, in the absence of direct analyses of endothelial integrity, we acknowledge that oxidative stress may play a role in the observed vascular reactivity, as it is a well-documented factor contributing to endothelial dysfunction in diabetes.

In the context of our experimental setting, although vascular grafts isolated from non-diabetic patients could be considered relatively healthy compared to the diabetic patients, it should be taken into account that all the patients included our study had coronary artery disease. Moreover, most patients had additional comorbidities such as hypertension and dyslipidemia independently of their diabetic and non-diabetic status. Therefore, we cannot completely rule out the contribution of other comorbidities influencing the vascular reactivity of bypass grafts. Supportively, a study involving hypertensive, hypercholesterolemic, and diabetic patients demonstrated that IMA rings harvested from hypertensive patients exhibited the greatest impairment in endothelium-dependent responses [37]. Therefore, a larger cohort study is needed to verify the changes in bypass graft reactivity and limit the effects of confounding factors.

Based on current trends, the rising incidence of diabetes will undoubtedly lead to increased cardiovascular mortality. It is well established that hyperglycemia leads to vascular hyperreactivity in diabetic patients [15] and animal models [38,39,40]. This effect may be attributed to endothelium-dependent and endothelium-independent mechanisms. In our study, increased responses to phenylephrine, an alpha-1 adrenoceptor agonist, both in IMAs and SVs, as well as increased responses to potassium chloride, a non-receptor agonist, in SVs from diabetic patients compared to non-diabetic patients, reinforced previous evidence suggesting increased vascular hyperreactivity in diabetes. Notably, our study is the first to demonstrate that diabetes exacerbates potassium chloride-induced contractions in isolated human SV grafts. Understanding the differences in potassium chloride-induced contraction profiles in arterial and venous grafts needs to be considered in order to optimize graft spasm and thus improve the patency rate of bypass grafts.

## 5. Conclusions

Our results indicate that diabetes enhances responses to phenylephrine, an alpha-1 adrenoceptor agonist, in both IMA and SV grafts and increases potassium chloride-induced contractions specifically in SV grafts. Under diabetic conditions, no deterioration of endothelium-dependent relaxations was observed in either isolated human SV or IMA grafts. This study is the first to show that diabetes exacerbates potassium chloride-induced contractions in isolated human SV grafts. Understanding these differences in contraction profiles is crucial in improving graft spasm management and bypass graft patency rates.

Our study could provide valuable insights into the functional characteristics of arterial and venous grafts in the context of diabetes, which could lead to several clinical benefits in terms alleviating graft spasm by tailor-made pharmacological treatments to improve overall outcomes and draw attention to the importance of diabetes management before and after surgery for maintaining superior long-term graft patency.

## Figures and Tables

**Table 1:** The demographic and perioperative characteristics of the patients.

	DM+	DM−	*p*-Value
Number of patients	12	10	
Sex			
Male/Female	10/2	10/0	
Age (years)	63.13 ± 2.5	58.38 ± 2.25	0.1801
IMA grafts	65.00 ± 3.29	55.25 ± 3.28	0.0805
SV grafts	61.25 ± 4.00	61.50 ± 2.53	0.9597
Pre-operative laboratory results			
Fasting blood glucose (mg/dL)	202.5 ± 20.70 ***	106.0 ± 4.31	0.0004
HbA1c %	8.87 ± 0.51 **	5.500 ± 0.37	0.0014
HDL (mg/dL)	44.50 ± 5.48	38.86 ± 3.68	0.3988
LDL (mg/dL)	100.5 ± 6.88	118.0 ± 15.33	0.3472
TG (mg/dL)	121.2 ± 11.24	175.6 ± 40.82	0.2575
Total cholesterol (mg/dL)	170.5 ± 6.80	178.9 ± 9.45	0.5013
Mean perioperative plasma glucose level (mg/dL)	200.3 ± 12.80 ***	128.4 ± 7.05	0.0002
HT	56%	50%	
Medications			
Aspirin	16%	26%	
ACE inhibitors	40%	26%	
ARBs	16%	16%	
Ca^+2^ channel blockers	4%	13%	
β-Blockers	20%	21%	
Statins	8%	3%	
Clopidogrel	12%	3%	
Insulin	40%		
Metformin	52%		
Gliclazide	24%		
Dapagliflozin	4%	3%	
Empagliflozin	4%		
Sitagliptin	4%		

All results are expressed as mean ± S.E.M. ** *p* < 0.01; *** *p* < 0.001.

**Table 2:** Potassium chloride (KCI)- and phenylephrine (Phe)-induced maximal contraction responses (Emax) and sensitivity (pEC50) to these spasmogenic agents in human internal mammary arteries (IMAs) and saphenous veins (SVs) isolated from diabetic (DM+) and non-diabetic (DM−) patients.

	Emax (g)	Emax (% of KCl 40 mM)	pEC50	n
KCl				
SV DM+	12.98 ± 1.83	113.8 ± 7.48 *	15.13 ± 7.94	5
SV DM−	7.92 ± 0.66	124.3 ± 4.17	11.17 ± 2.28	5
IMA DM+	5.46 ± 0.44	116.7 ± 1.97	23.83 ± 2.73	7
IMA DM−	4.32 ± 0.88	130.1 ± 8.03	24.53 ± 3.36	5
Phe				
SV DM+	10.33 ± 1.85	89.98 ± 9.53 *	5.69 ± 0.13	5
SV DM−	6.86 ± 0.63	108.3 ± 8.14	5.32 ± 0.15	5
IMA DM+	4.28 ± 0.27 ^##^	94.75 ± 8.79 ^###^	5.70 ± 0.12	7
IMA DM−	2.22 ± 0.33	75.61 ± 18.44	5.36 ± 0.16	5

All results are expressed as mean ± S.E.M. Emax values represent maximum contractions in “g” and % of KCl 40 mM. pEC50 values were calculated as −log EC50. * *p* < 0.05 SV DM+ vs. SV DM−. ^##^ *p* < 0.01; ^###^ *p* < 0.001 IMA DM+ vs. IMA DM−.

**Table 3:** Maximum relaxant responses (Emax) and pEC50 values of acetylcholine (ACh) and sodium nitroprusside (SNP) in human internal mammary arteries (IMAs) and saphenous veins (SVs) isolated from diabetic (DM+) and non-diabetic (DM−) patients.

	Emax (%)	pEC50	n
ACh			
SV DM+	30.9 ± 7.9	6.5 ± 0.1	5
SV DM−	26.2 ± 4.1	7.9 ± 0.7	5
IMA DM+	29.8 ± 3.9	7.0 ± 0.5	7
IMA DM−	39.0 ± 13.6	6.4 ± 0.1	5
SNP			
SV DM+	105.1 ± 5.4	9.2 ± 1.8	5
SV DM−	105.8 ± 1.7	7.4 ± 0.3	5
IMA DM+	105.0 ± 4.7	7.3 ± 0.1	7
IMA DM−	94.0 ± 12.2	6.7 ± 0.3	5

All results are expressed as mean ± S.E.M. Emax values represent maximum relaxations (% of phenylephrine precontraction). pEC50 values were calculated as −log EC50.

## Data Availability

We confirm that the main data supporting the findings of this study are available within the article, and any other additional data are available on request.

## Abbreviations

The following abbreviations are used in this manuscript:

CABG	coronary artery bypass grafting
IMA	internal mammary artery
SV	saphenous vein
DM	diabetes mellitus
DM+	diabetic
DM−	non-diabetic
KCl	potassium chloride
Phe	phenylephrine
ACh	acetylcholine
SNP	sodium nitroprusside
HbA1c	hemoglobin A1c
HDL	high-density lipoprotein
LDL	low-density lipoprotein
TG	triglyceride
HT	hypertension
ACE	angiotensin-converting enzyme
ARBs	angiotensin receptor blockers
S.E.M.	standard error of the mean
Emax	maximum effect
pEC50	Negative Logarithm of the EC50
RhoA	Ras Homolog Family Member A
NO	nitric oxide
